# Boosting Empathy and Compassion Through Mindfulness-Based and Socioemotional Dyadic Practice: Randomized Controlled Trial With App-Delivered Trainings

**DOI:** 10.2196/45027

**Published:** 2023-07-26

**Authors:** Sarita Silveira, Malvika Godara, Tania Singer

**Affiliations:** 1 Social Neuroscience Lab Max Planck Society Berlin Germany

**Keywords:** mental training, compassion, empathy, mindfulness, dyadic practice, acceptance, digital mental health, self-compassion, app-delivered training, Affect Dyad, mobile phone

## Abstract

**Background:**

Contemplative trainings have been found to effectively improve social skills such as empathy and compassion. However, there is a lack of research on the efficacy of app-delivered mindfulness-based and dyadic practices in boosting socioaffective capacity.

**Objective:**

The first aim of this study was to compare a novel app-delivered, partner-based socioemotional intervention (Affect Dyad) with mindfulness-based training to foster empathy and compassion for the self or others. The second aim of this study was to investigate the underlying mechanisms of these effects.

**Methods:**

This randomized controlled trial included socioemotional and mindfulness-based interventions and a waitlist control group, which received socioemotional training after the postintervention assessment. We used linear mixed-effects models to test intervention effects on self-report measures and an ecologically valid computer task of empathy, compassion for the self and others, and theory of mind. Moderated mediation models were used to investigate whether changes in acceptance, empathic distress, empathic listening, interoceptive awareness, and mindfulness served as underlying psychological processes of intervention effects.

**Results:**

In 218 participants (mean age 44.12, SD 11.71 years; 160/218, 73.4% female), we found all interventions to have positive effects on composite scores for compassion toward the self (β*_socioemotional_*=.44, *P*<.001; β*_waitlist socioemotional_*=.30, *P*=.002; β*_mindfulness-based_*=.35, *P*<.001) and others (β*_socioemotional_*=.24, *P*=.003; β*_waitlist socioemotional_*=.35, *P*<.001; β*_mindfulness-based_*=.29, *P*<.001). Compassion measured with the computer task did not change significantly but showed a trend toward increase only in socioemotional dyadic practice (β*_socioemotional_*=.08, *P*=.08; β*_waitlist socioemotional_*=.11, *P*=.06). Similarly, on the empathic concern subscale of the Interpersonal Reactivity Index, a nonsignificant trend toward increase was found in the socioemotional intervention group (β*_socioemotional_*=.17; *P*=.08). Empathy significantly increased in both socioemotional groups (β*_socioemotional_*=.16, *P*=.03; β*_waitlist socioemotional_*=.35, *P*<.001) and the mindfulness-based group (β*_mindfulness-based_*=.15; *P*=.04). The measures of theory of mind did not change over time. In the mindfulness-based group, the increase in self-compassion was mediated by a decrease in empathic distress (indirect effect *ab_mindfulness-based_*=0.07, 95% CI 0.02-0.14). In the socioemotional group, an increase in self-compassion could be predicted by an increase in acceptance (β*_socioemotional_*=6.63, 95% CI 0.52-12.38).

**Conclusions:**

Using a multimethod approach, this study shows that app-delivered socioemotional and mindfulness-based trainings are effective in fostering compassion for the self and others in self-report. Both low-dose trainings could boost behavioral empathy markers; however, the effects on behavioral and dispositional markers of compassion only trended after dyadic practice, yet these effects did not reach statistical significance. Training-related increases in self-compassion rely on differential psychological processes, that is, on improved empathic distress regulation through mindfulness-based training and the activation of a human care– and acceptance-based system through socioemotional dyadic training.

**Trial Registration:**

ClinicalTrials.gov NCT04889508; https://clinicaltrials.gov/ct2/show/NCT04889508

## Introduction

### Background

In the past decades, there has been growing evidence for the beneficial effects of contemplative trainings on mental and physical health [1-3] and the development of social skills such as empathy, compassion, and prosocial motivation and behavior [4-6]. However, most of these secular mindfulness or compassion trainings, such as the popular 8-week Mindfulness-Based Stress Reduction program [7], the 8-week Mindful Self-Compassion program [8], or compassion-focused therapy [9], require in-person individual or group sessions.

Recently, and especially in times of the COVID-19 pandemic, considerable awareness has been raised regarding digital mental health solutions to prevailing public health burdens [10,11]. Social emotions such as empathy and compassion have been identified as psychological factors related to mental health resilience [12,13]. In particular, the cultivation of compassion has been called to action to mitigate the impact of the pandemic on psychological well-being [14]. Smartphone-delivered contemplative practices might be promising tools for this endeavor [15,16]. However, such mental trainings are diverse in both the focus and modality of practice. On the one hand, they can focus on cultivating attention-related, socioemotional, or meta-cognitive capacities (for a review of the differential effects of type of practice, see the study by Singer and Engert [1]). In contrast, although most mindfulness-based practices are solitary, more recent approaches have included interpersonal practice [17-19]. The differential effects of the modalities of app-delivered practice and their underlying mechanisms are largely unknown. Therefore, in this randomized controlled trial (RCT), we compared a novel, partner-based socioemotional practice, the so-called Affect Dyad [19], with mindfulness-based practice in its efficacy in increasing empathy and self- or other-related compassion using a multimethod approach. These 10-week trainings were performed in the context of a COVID-19–related mental health study, the CovSocial project [20].

### Empathy and Compassion

As a social species, humans are equipped with affective and cognitive social capacities that enable an understanding of the feelings, mental states, and intentions of others [21,22]. An important socioaffective capacity is empathy, which describes the ability to share another’s affective state while being aware that its source is the other person [23]. Empathy allows an individual to share both unpleasant and pleasant emotions with others and is ingrained in our neurophysiology [24]. Although empathy helps us better understand one another and foster social cohesion [23], it can also turn into so-called empathic or personal distress when confronted with intense negative feelings or when the self-other distinction fails [25,26]. Empathic distress is a self-related aversive state that is associated with the wish to withdraw and alleviate one’s own negative feelings [25,26]. It presumably is a cause of the observed high burnout rates in health care providers [27,28], a problem that was exacerbated during the COVID-19 pandemic [29]. Compassion and self-compassion have been proposed as protective factors against such negative consequences [26,30].

In contrast to empathy, compassion is characterized by a concern for others, often associated with feelings of warmth and care, and the wish to alleviate their negative feelings [26]. Rather than *feeling with*, compassion is *feeling for* a person and, as such, not an emotion per se but a motivation to help others [9,31]. Thus, compassion can be referred to as altruistic other-related capacity and has long been proposed as a crucial driver of prosocial behaviors [32-34]. Indeed, empathic and compassionate responses to others differ on a neurophysiological level, with empathy relying on a brain network associated with pain [24,35] and compassion relying on a brain network associated with positive feelings and reward [26,35,36].

In the past decades, compassion has been conceptually divided into self- and other-related components [8]. Self-compassion has gained extensive attention in resilience research as a means of alleviating distress and promoting well-being [37-39]. The distinction between self- and other-related compassion has shaped contemplative practice and research. Practices such as loving-kindness meditation incorporate successive training of kindness toward the self and others [40,41]. Similarly, questionnaires have been developed to measure both aspects [42,43]. However, most intervention studies to date have focused on either aspect, with a disproportionate focus on self-compassion [44,45]. This study systematically compared 2 different practice types in their effects on both aspects of compassion using a multimethod approach. More specifically, and because of the low reliability of some self-report scales [46], we used a factor analytical approach across multiple scales of compassion for the self and others to derive more reliable higher-level composites of the 2 components of compassion.

### Differences in Contemplative Practice

A main goal of this RCT was to compare the efficacy of 2 contemplative practice types that differ in content and modality. Generally speaking, mindfulness-based interventions focus on present-moment awareness and attention regulation of thoughts, feelings, or body sensations [47]. With increasing research on trainings that focus on compassion, such as the Mindful Self-Compassion program [8] or compassion-focused therapy [9], debates about the differential efficacy of mindfulness-based and compassion trainings have started to grow.

The findings of a large-scale 9-month mental training study, the ReSource project [41], provide evidence that the type and content of the respective practice matter for a large variety of outcomes [1]. With regard to social emotions, the socioemotional Affect module, which focuses on care, gratefulness, loving-kindness, and acceptance, outperformed the 2 other training modules in increasing other-related compassion in self-report [48] and the computer-based EmpaToM task [49]. Similarly, other studies have shown that, although both mindfulness-based and compassion-based interventions confer benefits on self-compassion, compassion-based approaches have an advantage in promoting compassion toward others [44,50]. In the ReSource project, the sociocognitive Perspective module, with a focus on meta-cognitive abilities and perspective taking, rather than the compassion-based Affect module, could specifically improve theory of mind (ToM) [4]. In contrast to the socioaffective capacities of empathy and compassion, ToM, which is also referred to as perspective-taking or mentalizing [51], is a sociocognitive skill that enables an understanding of others through inference about their thoughts and beliefs [52].

Apart from differences in content (eg, attention, compassion, and meta-cognition), contemplative practices also differ in modality. In contrast to solitary mindfulness-based practice, intersubjective practice formats have recently gained attention [17-19]. Intersubjective mindfulness-based interventions showed additional benefits for self-compassion, empathy, and reduced empathic distress in health care professionals [53]. In dyad practice, 2 individuals are paired in a guided contemplative conversation with switching roles of speaker and listener [19]. Although it has previously been shown that different types of dyads can promote social connectedness and social disclosure [19], the intersubjective format of dyad practice has not yet been compared with solitary practice. In this study, we aimed to test whether (1) low-dose web-based trainings with daily Affect Dyad or mindfulness-based practice could boost social emotions and (2) the Affect Dyad could outperform solitary mindfulness-based practice in improving empathy and compassion because of its explicit focus on cultivating socioaffective capacities such as empathic listening and the acceptance of difficult emotions in the presence of another person.

### Mechanisms of Contemplative Practice

Finally, our last goal was to gain insights into the psychological processes that underlie the effects of contemplative practices. Despite growing research interest in contemplative trainings, a mechanistic understanding of practice effects is still scarce. According to a current classification system, distinct cognitive mechanisms are relevant to differential practice effects [54]. Thus, the effects of mindfulness-based practice relate to improvements in self-related processes [54-56] such as emotion regulation strategies [57,58], equanimity (ie, a balanced reaction to emotions) [59,60], or embodied cognition and interoceptive awareness [58,61,62]. In particular, the latter is associated with the development of affect-sharing abilities as supported by common neurofunctional correlates [63].

In contrast, loving-kindness and compassion-based practice is proposed to rely on an active generation of positive affect, acceptance, and prosocial motivation [64]. Although the Affect Dyad [19], unlike loving-kindness meditation, does not explicitly train compassion for self and others, it is expected to activate the motivational-biological care system [1,26]. This care system promotes social bonds and feelings of nurture, love, and acceptance [65]. Interestingly, nonjudgmental attitude and acceptance, which are conceptualized as aspects of mindfulness [47], were found to relate to feelings of warmth and care and to be promoted by compassion-based rather than mindfulness-based trainings [48,66,67]. Therefore, we aimed to test whether different psychological processes may underlie the observed training effects.

### Study Goals

The first aim of this RCT was to compare the effects of 2 brief app-delivered practice types. Specifically, attention-based, solitary mindfulness-based practices were compared with a novel socioemotional dyadic practice, Affect Dyad [19], on different outcome measures of empathy and compassion for the self and others. Using a multimethod approach, we investigated differential practice effects on validated state and trait questionnaires as well as on an ecologically valid computer task, the EmpaToM [49]. Furthermore, we aimed to investigate the different psychological processes underlying these practice effects. To this effect, in addition to pre- and postintervention assessments, the study design included a weekly assessment of a variety of mediator variables over the 10-week training duration in both intervention groups.

In our preregistered hypotheses [68], we expected that socioemotional dyadic practice would increase compassion significantly more than mindfulness-based practice in both self-report measures and computer-based tasks. We further expected that both trainings would lead to an increase in empathy compared with that in the waitlist control group. The inclusion of sociocognitive ToM performance served as a control condition. As neither practice focused on perspective-taking, no intervention-related change in ToM was expected (see also the study by Trautwein et al [4]).

With regard to the underlying processes of practice effects, we expected that, in the socioemotional training, an increase in compassion would be mediated by socioaffective and motivational processes such as an increase in acceptance and empathic listening and a decrease in empathic distress. Thus, changes in these mediators over time were expected to be significantly associated with changes in compassion from before to after the intervention. We further expected an increase in empathy to be mediated by an increase in mindfulness and interoceptive awareness. Thus, changes in these mediators over time were expected to be significantly associated with changes in empathy from before to after the intervention.

## Methods

### Sample Recruitment

As part of the CovSocial project [20], a longitudinal study on psychological vulnerability, resilience, and social cohesion during the COVID-19 pandemic in 2020 and 2021 in Berlin, 3522 participants from the first project phase, that is, the main study sample in phase 1, were invited to complete a prescreening procedure. With regard to exclusion criteria, participants had to be naive to meditation and yoga practices; have no educational background in psychology; have no current psychopathology, suicidality, chronic illness, or pain; and not use illicit or prescribed substances that affect physiological stress markers.

After the information webinars (see *Welcome Days* in Multimedia Appendix 1 [19,41]), a senior researcher in the project randomized participants into 3 groups—2 intervention groups and 1 waitlist control group using computer-generated numbers in a lock randomization technique with 1:1:1 allocation. Intervention arms were assigned to participants by the study coordinator. Screening calls were conducted by 4 trained meditation teachers (see *Teacher Training* in Multimedia Appendix 1) to exclude individuals with clinical levels of psychopathology using the Standardized Assessment of Severity of Personality Disorder [69] and Composite International Diagnostic Screener [70].

**Figure 1:**
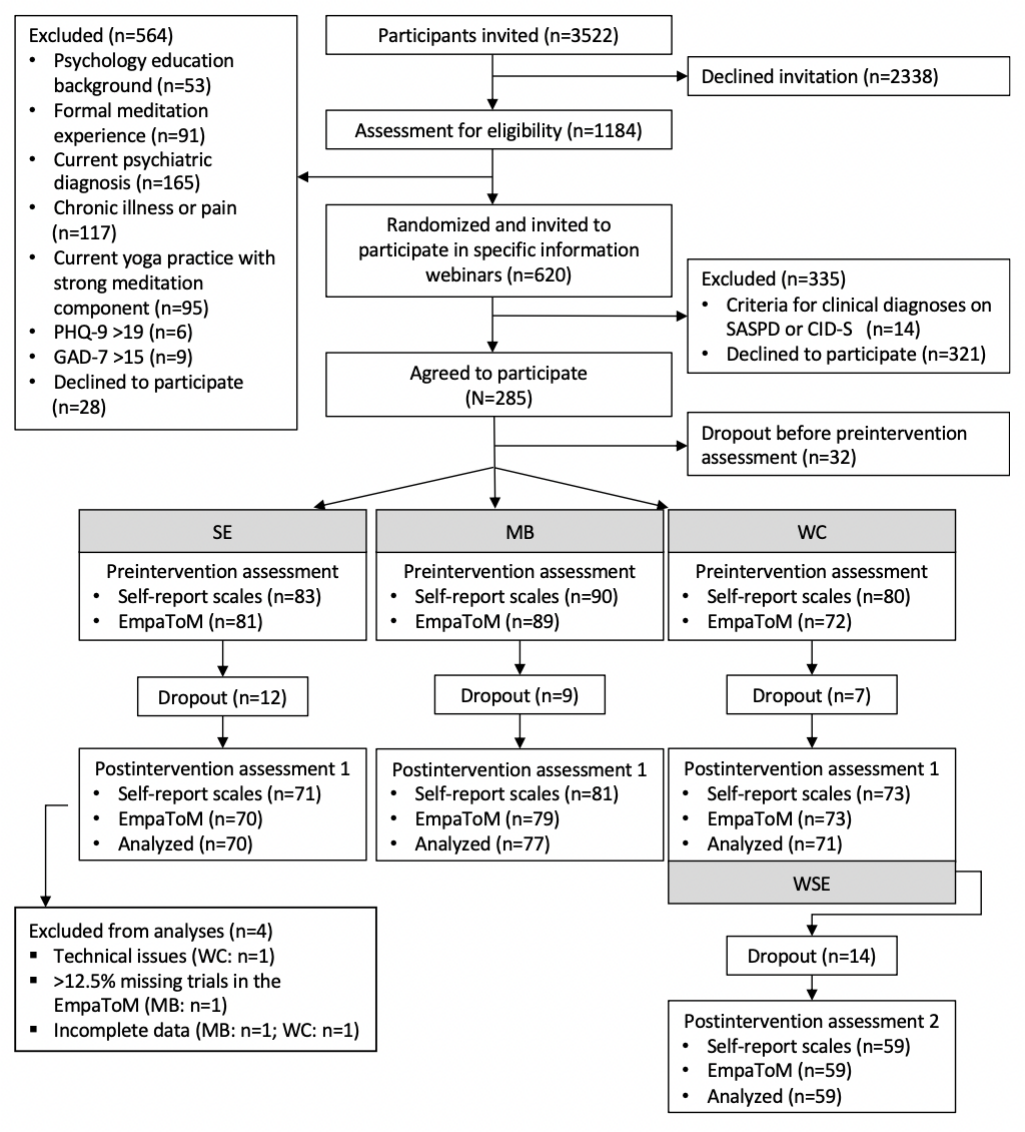
Flow diagram of sample selection for the randomized controlled trial of the CovSocial project phase 2. CID-S: Composite International Diagnostic Screener; GAD-7: General Anxiety Disorder–7; MB: mindfulness-based; PHQ-9: Patient Health Questionnaire–9; SASPD: Standardized Assessment of Severity of Personality Disorder; SE: socioemotional; WC: waitlist control; WSE: waitlist socioemotional.

A total of 285 participants registered for the RCT, which was in line with the a priori determined sample size (Multimedia Appendix 2 [41,71]). With respect to sociodemographic variables, there was some selection bias toward more female participants and participants with higher levels of education and income compared with the remaining phase 1 participants of the CovSocial project and the Berlin general population (Multimedia Appendix 3 [72]). This study reported on 218 participants (Figure 1 and Table 1). All study participants provided written informed consent. A financial compensation of €10 (US $10.70) per hour was offered.

**Table 1:** Sample demographics split by intervention group (n=218).

			SE^a^ (n=70)		MB^b^ (n=77)		WC^c^ or WSE^d^ (n=71)
Age (years), mean (SD)			43.19 (11.98)		43.64 (11.64)		45.58 (11.58)
**Sex, n (%)**							
	Male	16 (23)		24 (31)		18 (25)	
	Female	54 (77)		53 (69)		53 (75)	
**Marital status, n (%)**							
	Single	46 (66)		49 (64)		44 (62)	
	Married or cohabiting	24 (34)		28 (36)		27 (38)	
Years of education, mean (SD)			18.27 (4.11)		16.99 (3.41)		18.51 (3.17)
**Employment status, n (%)**							
	Full time or part time	54 (77)		67 (87)		61 (86)	
	None	16 (23)		10 (13)		10 (14)	
**Household income, n (%)**							
	Above average^e^	44 (63)		52 (68)		51 (72)	
	Below average^e^	26 (37)		25 (32)		20 (28)	
Lifetime prevalence of mental disorder, n (%)			16 (23)		11 (14)		16 (23)

^a^SE: socioemotional intervention.

^b^MB: mindfulness-based intervention.

^c^WC: waitlist control.

^d^WSE: waitlist socioemotional intervention.

^e^The average monthly net income in Berlin is approximately €2175 (US $2327.03) [72].

### Study Design

#### Overview

**Figure 2:**
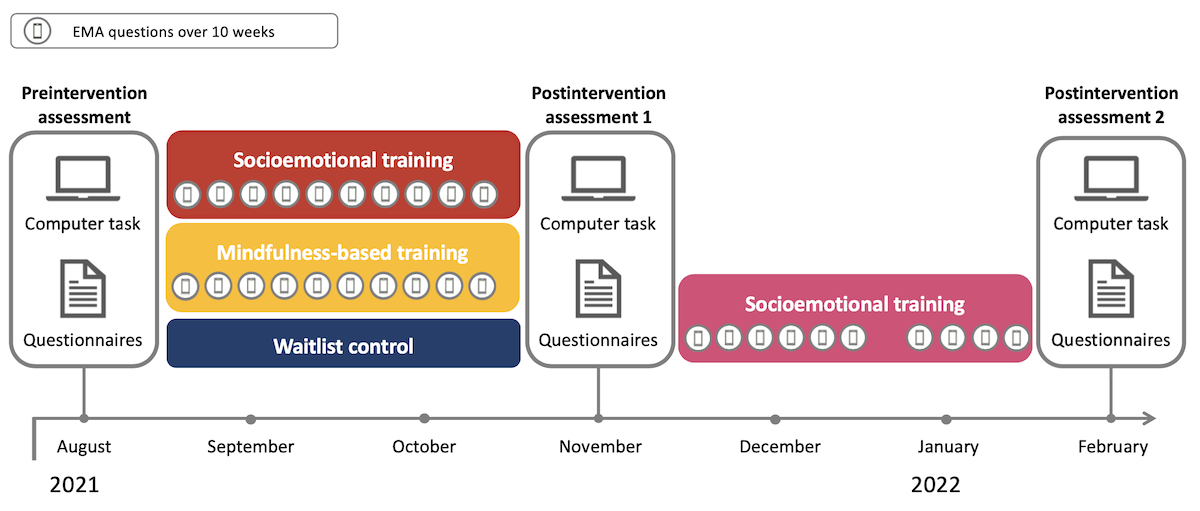
Study design of the CovSocial phase 2, including preintervention and postintervention assessment as well as weekly study measures. EMA: ecological momentary assessment.

This paper reports on data from an RCT (trial registration: ClinicalTrials.gov NCT04889508), which builds the second phase of the CovSocial project (Figure 2). The first intervention interval took place between August 2021 (preintervention assessment) and November 2021 (postintervention assessment 1) and included the socioemotional and mindfulness-based intervention groups and a waitlist control group. The waitlist control group underwent the socioemotional intervention after postintervention assessment 1, with an additional postintervention assessment 2 in February 2022 and March 2022. The intervention began with a web-based kick-off event before preintervention assessment 1 and 2 web-based onboarding sessions simultaneously with preintervention assessment (Multimedia Appendix 1).

The study design and analyses were preregistered [68]. There were 2 deviations from the preregistration concerning the variables included in this study. Weekly assessed items of self-kindness (1 item on the long form of the State Self-Compassion Scale [SSCS-L]; [73]) and fear of compassion (2 items on the Fears of Compassion Scales [FoC]; [42]) were not included in the analyses as these items are now included in the composites for self- and other-related compassion at preintervention assessment, postintervention assessment 1, and postintervention assessment 2. The use of composite scores for self- and other-related compassion was not preregistered despite being an aim of this study to increase the reliability of constructs through a factor analytical approach given that single scales have been criticized for having low reliability [46]. In addition, in the analyses in this study, we had to refrain from including further daily assessed variables that might function as mediators of change in both intervention groups as these variables will be used in other papers of the CovSocial project phase 2, which are currently in preparation.

#### Socioemotional Intervention

The partner-based socioemotional intervention consisted of 10 weeks of practice of a daily dyadic exercise with a randomly assigned partner, the so-called Affect Dyad. Daily practice of the Affect Dyad begins with a moment of silence wherein participants are required to center themselves and let go of any current thoughts and feelings. After this, the first partner speaks about a difficult or stressful situation that they experienced during the past 24 hours and how emotions that occurred during that situation felt in their body. While the first partner speaks, the other partner remains silent and is instructed to listen in a nonjudgmental and empathic manner without providing any verbal or nonverbal communication. After speaking for 2.5 minutes about the difficult situation, the first partner then speaks about a situation in the past 24 hours that made them feel grateful and how the experience of gratitude felt in their body. The listener again remains silent and listens with an empathic and nonjudgmental stance. Once the first partner finishes speaking about the grateful situation for 2.5 minutes, and after another 30 seconds of silence, the procedure is then repeated with the roles of speaker and listener switched. The exercise ends with another moment of silence. Participants were instructed to perform this daily exercise 6 times a week. Before each week of daily dyad practice, the participants were randomly paired with a new participant in the socioemotional intervention group. The goal of the Affect Dyad exercise is to enhance coping with difficult emotions through acceptance and increase social connectedness, acceptance of self and others, empathic and nonjudgmental listening, and gratitude.

#### Mindfulness-Based Intervention

The mindfulness-based intervention consisted of 10 weeks of individual attention-focused mindfulness meditation practice. One of the core practices was breathing meditation, which is a 12.5-minute individual exercise that requires participants to focus their attention on the sensations of breathing. Participants have to sustain their attention on their breath for long stretches of time. When their minds wander, participants are instructed to return their attention to their breath. Participants also engaged in other practices, such as attention-based mindfulness of sounds (the object of attention are sounds in the environment) and open-presence meditation (the object of attention are sensations present in the inner and outer environment). Daily practice of the meditation was guided by prerecorded audios. The exercise begins with participants being asked to sit in a comfortable position that makes them relaxed yet keeps them awake and aware. They are asked to focus on the sensation of their current body placement and position and cultivate an attitude of dignity and receptivity toward themselves and their bodies. The key focus of these practices is on training present-moment attention and interoceptive body awareness.

#### Coaching Sessions

Daily practice took place 6 days per week using the CovSocial app (Multimedia Appendix 4). It was supported by weekly 2-hour web-based coaching sessions with 1 of 4 meditation teachers to help deepen practice effects and anchor training in everyday life (Multimedia Appendix 4). The coaching sessions covered the following topics for socioemotional training: dyad ritual, body language, empathic listening, gratitude, dealing with difficult emotions, recognizing patterns in life, and the transfer of the dyad experience to daily life. The coaching sessions covered the following topics for mindfulness-based training: basics of breathing meditation, body awareness, sensory perceptions, engaging all 5 senses, open awareness, dealing with stress, and the transfer of meditation practice to daily life. The weekly coaching combined short presentations with guided group discussions as well as conversations in breakout rooms that focused on individual experiences. The content of the presentations was specific to the respective intervention [20] (Multimedia Appendix 1).

### Measures

Self-report and behavioral measures were taken at preintervention assessment and postintervention assessment 1 in the socioemotional, mindfulness-based, and waitlist control groups and at postintervention assessment 2 in the waitlist socioemotional group. In addition, several variables were assessed on a weekly basis in all intervention groups using push notifications on the project’s smartphone app to trace the potential mechanisms of the intervention effects (Figure 2).

#### Compassion Scales

Self-compassion was assessed using the long form of the SSCS-L [73]. The SSCS-L consists of 18 items, with 3 items each constituting 1 of 6 subscales. Half of the subscales represent negative components, and the other half represent positive components of self-compassion. Components include self-judgment (eg, “I’m being pretty tough on myself.”) versus self-kindness (eg, “I’m being supportive toward myself.”), isolation (eg, “I feel separate and cut off from the rest of the world.”) versus common humanity (eg, “I see my difficulties as part of life that everyone goes through.”), and overidentification (eg, “I’m getting carried away with my feelings.”) versus mindfulness (eg, “I’m keeping things in perspective.”). Items are rated on a 5-point scale ranging from “almost never” (1) to “almost always” (5).

To assess compassion in a dualistic manner as divided into self- and other-related compassion, we used the Sussex-Oxford Compassion for Others Scale and the Sussex-Oxford Compassion for the Self Scale (SOCS-S) [43]. Both scales consist of 20 items, with 5 subscales that consist of 4 items each. The subscales capture different aspects of self- and other-related compassion, including recognizing suffering (eg, “I notice when others are [I am] feeling distressed.”), understanding the universality of suffering (eg, “I understand that feeling upset at times is part of human nature.”), feeling for the person suffering (eg, “When someone is [I am] upset, I try to tune in to how they’re [I’m] feeling.”), tolerating uncomfortable feelings (eg, “When someone else is [I’m] upset, I try to stay open to their [my] feelings rather than avoid them.”), and acting or being motivated to act to alleviate suffering (eg, “When I see someone in need [When I’m upset], I try to do what’s best for them [myself].”). Items are rated on a 5-point scale ranging from “not at all true” (1) to “always true” (5).

In total, 2 subscales of the FoC [42] were used to assess beliefs that prevent individuals from experiencing or expressing compassion. The subscales include 15 items regarding expressing kindness and compassion toward oneself (eg, “I feel that I don’t deserve to be kind and forgiving to myself.”) and 10 items regarding expressing compassion for others (eg, “People will take advantage of me if they see me as too compassionate.”). The 5-point rating scale ranges from “don’t agree at all” (0) to “completely agree” (4).

#### EmpaToM

To assess socioaffective and sociocognitive performance in a behavioral task, we also used the computer-based EmpaToM paradigm [49]. Each trial starts with a short video clip (approximately 15 seconds) with autobiographical narratives performed by actors and of either emotionally neutral or negative content. After each video, participants are first asked how they felt on a rating scale from “negative” (−4) to “positive” (4). Second, participants are asked to rate how much compassion they felt, from “none” (0) to “very much” (8). Third, a question that demands either ToM inference or factual reasoning on the video’s content is presented in a multiple-choice response format for a maximum of 14 seconds. The EmpaToM consists of 48 trials in a 2 (neutral or negative) × 2 (ToM or no ToM) factorial design, with 12 videos of the same 12 actors per condition to control for actor-specific effects. For repeated testing, different parallel sets of stimuli were presented to participants at preintervention assessment, postintervention assessment 1, and postintervention assessment 2, which were matched and validated for repeated testing of empathy, compassion, and ToM [74].

#### Interpersonal Reactivity Index

In total, 2 subscales for empathic concern (eg, “When I see someone being taken advantage of, I feel kind of protective towards them.”) and perspective taking (eg, “Before criticizing somebody, I try to imagine how I would feel if I were in their place.”) of the German version [75] of the Interpersonal Reactivity Index (IRI) [51] were used as a self-report measure that differentiates between socioaffective and sociocognitive skills. Each subscale consists of 4 items on a 5-point rating scale from “never” (1) to “always” (5).

#### Mediator Variables

To assess acceptance, 1 item on the Cognitive Emotion Regulation Questionnaire [76] was used (“I accept difficult situations in my life”). Empathic listening was measured using 1 self-generated item (“[in the past week] How well were you able to listen to another person during social interactions?”). Empathic distress was assessed using an item on the personal distress subscale of the IRI [75] (“Being in a tense emotional situation scares me.”). Mindfulness was measured using 5 items on the Five Facet Mindfulness Questionnaire [77] that corresponded to the questionnaire’s 5 subscales (ie, “I’m good at finding the words to describe my feelings,” “I pay attention to sensations, such as the wind in my hair or sun on my face,” “I rush through activities without being really attentive to them,” “I tell myself I shouldn’t be thinking the way I’m thinking,” and “I watch my feelings without getting lost in them”). Interoceptive awareness was assessed using 2 items on the Multidimensional Assessment of Interoceptive Awareness [78]. Although the Multidimensional Assessment of Interoceptive Awareness entails a total of 8 subscales, only self-regulation (ie, “I can calm my mind by focusing on my body and breathing”) and body listening (“I listen for information from my body about my emotional state”) were assessed as previous research suggests that these aspects of interoceptive awareness can be promoted through contemplative training [62]. All items were rated on a 5-point scale from “not at all” (0) to “very much” (4).

### Statistical Analysis

#### Factor Analyses and Scale Preparation

All statistical data analyses were performed using R (R Foundation for Statistical Computing) [79]. To determine whether self-report scales on compassion can be grouped into 2 composites for compassion toward the self and others, split-sample (n*_train_*=110) exploratory and confirmatory factor analyses were computed. Postintervention-assessment-2 data were excluded from these analyses because of insufficient sample size. Parallel analyses with 1000 iterations and eigenvalues of >1 were used to determine the number of factors at each measurement occasion. Exploratory factor analyses used Promax factor rotation. Subsequent confirmatory factor analysis included constraints of scalar measurement invariance across preintervention assessment and postintervention assessment 1 and robust maximum likelihood estimation. Fit indexes of root mean square error of approximation (RMSEA), comparative fit index, and Tucker-Lewis index (TLI) as well as chi-square statistics and *df* are reported.

Scores for empathy were calculated by subtracting the affect ratings after neutral videos from those after negative videos. In line with previous findings that compassion training is not constrained to situations of negative feelings [4,35], compassion scores were computed by averaging across the negative and neutral conditions. ToM scores were computed by averaging the standardized error rates and response times in ToM trials [49]. Please see Multimedia Appendix 5 for the descriptive statistics for ToM accuracy and response times.

#### Change Analyses

To investigate intervention effects on self-report (ie, factor scores derived from composites for self- and other-related compassion and the IRI subscales of empathic concern and perspective taking) and behavioral measures (ie, EmpaToM scores for compassion, empathy, and ToM), linear mixed-effects models (LMMs) were computed. The scores of each dependent variable were standardized by their overall SD to allow for comparability. LMMs included random intercepts for participants and fixed effects for group, time, and their interaction term, with waitlist control defined as the reference group and backward difference coding for the time factor. Participants’ sex and age were included as covariates. Planned contrasts (ie, socioemotional–waitlist control, mindfulness-based–waitlist control, waitlist socioemotional–waitlist control, and socioemotional–mindfulness-based) were computed. Model estimates of planned contrasts were used as effect size estimates, which were classified in accordance with standard conventions (ie, small: ≥0.20; medium: ≥0.50; large: ≥0.80). Theory-driven contrasts were not corrected for multiple testing.

To test for an association of behavioral measures of compassion and ToM (EmpaToM) with self-report measures of empathic concern and perspective taking (IRI), as well as with composites for self- and other-related compassion, Spearman correlation coefficients were computed using data from the study preintervention assessment. The results of these analyses are reported in Multimedia Appendix 6 [49,51].

#### Moderated Mediation Analyses

As a first step, LMMs were used to investigate intervention effects on longitudinal changes in the weekly measured variables of acceptance, empathic listening, empathic distress, mindfulness, and interoceptive awareness. For mindfulness and interoceptive awareness, mean scores were computed across the corresponding items. In the first week of the first intervention phase, data on empathic listening were missing for 66.5% (145/218) of the participants because of technical issues with the smartphone app. Therefore, analyses of longitudinal changes in empathic listening only included weeks 2 to 10. Participants with data on <3 measurement occasions were excluded from longitudinal change and mediation analyses (3/218, 1.4%). As dependent measures were only assessed in the intervention groups, the main models included only the socioemotional and mindfulness-based groups, with fixed effects for intervention group, time, and an interaction term of group and time and with mindfulness-based defined as the reference group. The models included random intercepts and slopes. The separate random-intercept models for the waitlist socioemotional group in the second intervention phase included a fixed effect of week. The estimated individual slope coefficients were extracted from the LMMs for further mediation analyses.

In the mediation analyses, for changes from preintervention assessment to postintervention assessment 1 in each self-report and behavioral outcome measure of compassion for the self and others and empathy (*c-path*), we tested whether the slopes of acceptance, empathic listening, empathic distress, mindfulness, and interoceptive awareness served as mediators of the effect and whether the association between slope and outcome (*b-path*) and the direct effect of change (*c’-path*) was moderated by the intervention group. Continuous slope variables were centralized. Sex and age were included as covariates. Each model used 5000 bootstrap iterations. Bootstrap CIs were reported.

### Ethics Approval

This study was conducted in accordance with the Declaration of Helsinki and was approved by the ethics committee of the Charité – Universitätsmedizin Berlin, Germany (EA4/081/21).

## Results

### Outcome Evaluation

#### Self-Compassion and Other-Related Compassion

**Figure 3:**
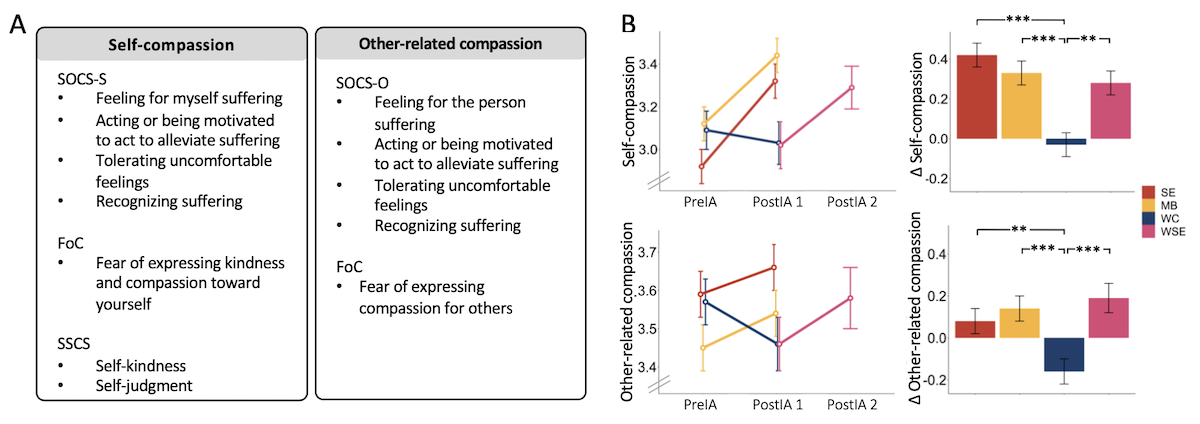
(A) Composites of self-compassion and other-related compassion; (B) mean plots of those composites in the socioemotional (SE), waitlist socioemotional (WSE), and mindfulness-based (MB) intervention groups and the waitlist control (WC) group; and group differences in intervention-related change. Means and standard errors; significance level of *P=.05, **P=.01, and ***P=.001. FoC: Fears of Compassion Scales; SOCS-O: Sussex-Oxford Compassion for Others Scale; SOCS-S: Sussex-Oxford Compassion for the Self Scale; SSCS: State Self-Compassion Scale; PreIA: preintervention assessment; PostIA: postintervention assessment.

For exploratory factor analyses of self-report measures of compassion, parallel analyses suggested 4 factors at preintervention assessment and postintervention assessment 1. In the training sample, the 4-factor solution had an acceptable fit at preintervention assessment (TLI=0.90; RMSEA=0.075, 95% CI 0.050-0.100) and a fit of TLI=0.83 and RMSEA=0.103 (95% CI 0.082-0.126) at postintervention assessment. The 2 main factors could be confirmed in the test sample with scalar measurement invariance (*χ*^2^_276_=410.6, *P*<.001; comparative fit index=0.91; TLI=0.90; RMSEA=0.074, 95% CI 0.060-0.087). The first factor consisted of all SOCS-S subscales except for understanding suffering, as well as the inverted FoC scale for the self and the 2 SCSS-L subscales of self-kindness and self-judgment, with an internal reliability of α*_preintervention assessment_*=.88, α*_postintervention assessment 1_*=.88, and α*_postintervention assessment 2_*=.88. The second factor consisted of all Sussex-Oxford Compassion for Others Scale subscales except for understanding suffering and the inverted FoC scale for others, with an internal reliability of α*_preintervention assessment_*=.81, α*_postintervention assessment 1_*=.83, and α*_postintervention assessment 2_*=.87 (Figure 3A). Standardized composites for self- and other-related compassion were built from these indicators, respectively.

In the LMM on self-compassion, both partner-based socioemotional and waitlist socioemotional trainings (β*_socioemotional_*=.44, *P*<.001; β*_waitlist socioemotional_*=.30, *P*=.002) and the solitary mindfulness-based training (β*_mindfulness-based_*=.35; *P*<.001) showed small effects in increasing self-compassion compared with the waitlist control group. No differential effect was found between the socioemotional and mindfulness-based intervention groups (β*_differentiation_*=.09; *P*=.14).

For other-related compassion, again, small intervention effects could be found for both partner-based mental trainings (β*_socioemotional_*=.24, *P*=.003; β*_waitlist socioemotional_*=.35, *P*<.001) and the mindfulness-based training (β*_mindfulness-based_*=.29; *P*<.001) in increasing other-related compassion compared with the waitlist control group. When compared with each other, the socioemotional and mindfulness-based intervention groups did not show differential effects (β*_differentiation_*=−.09; *P*=.14; Figure 3B).

#### EmpaToM

**Figure 4:**
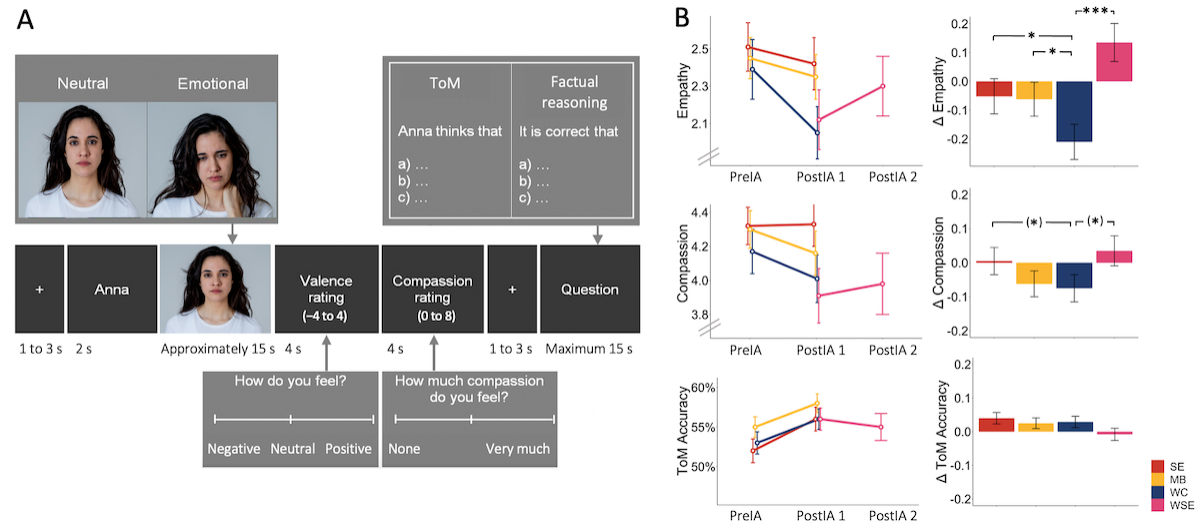
(A) Schematic illustration of an EmpaToM trial sequence to assess empathy, compassion, and theory of mind (ToM). (B) Mean plots of empathy, compassion, and ToM in the socioemotional (SE), waitlist socioemotional (WSE), and mindfulness-based (MB) intervention groups and the waitlist control (WC) group and group differences in intervention-related change. Means and standard errors; significance level of (*) .10>*P*>.05, *P=.05, and ***P=.001. PreIA: preintervention assessment; PostIA: postintervention assessment. Picture source: sbartsmediagmail.com/Shotshop.com.

In the computer task (Figure 4A), the intervention effects on an increase in empathy were very small during the first intervention period in both the socioemotional (β*_socioemotional_*=.16; *P*=.03) and mindfulness-based (β*_mindfulness-based_*=.15; *P*=.04) mental trainings. Small effects were found in the second partner-based waitlist socioemotional group (β*_waitlist socioemotional_*=.35; *P*<.001). There was no difference between socioemotional and mindfulness-based intervention effects (β*_differentiation_*=.01; *P*=.45).

With regard to compassion as measured using the EmpaToM, although none of the intervention effects reached significance, there was a trend toward an increase in compassion only in the 2 partner-based training groups (β*_socioemotional_*=.08, *P*=.08; β*_waitlist socioemotional_*=.11, *P*=.06) compared with the waitlist control group. No effect was found for the mindfulness-based mental training group (β*_mindfulness-based_*=.01; *P*=.41) or for a differentiation between the mental training conditions (β*_differentiation_*=.07; *P*=.11).

None of the interventions had an effect on ToM from preintervention to postintervention assessment compared with the waitlist control condition (β*_socioemotional_*=−.01, *P*=.91; β*_mindfulness-based_*=.04, *P*=.74; β*_waitlist socioemotional_*=.09, *P*=.52), and there was no differential effect between the mindfulness-based and socioemotional groups (β*_differentiation_*=−.05; *P*=.66; Figure 4B).

#### IRI Results

In the LMM on empathic concern, although there was no significant intervention effect in any of the groups (β*_mindfulness-based_*=.09, *P*=.23; β*_waitlist socioemotional_*=.15, *P*=.17) and no differential effect between the mindfulness-based and socioemotional training conditions (β*_differentiation_*=.08; *P*=.25), a trend toward an increase in empathic concern was found for the partner-based mental training in the first intervention period (β*_socioemotional_*=.17; *P*=.08). There were no intervention effects on perspective taking in any of the intervention groups (β*_socioemotional_*=.10, *P*=.52; β*_mindfulness-based_*=−.07, *P*=.62; β*_waitlist socioemotional_*=−.24, *P*=.20) and no differential effects between the socioemotional and mindfulness-based conditions (β*_differentiation_*=.17; *P*=.24; Multimedia Appendix 7).

### Mechanisms of Intervention Effects

**Figure 5:**
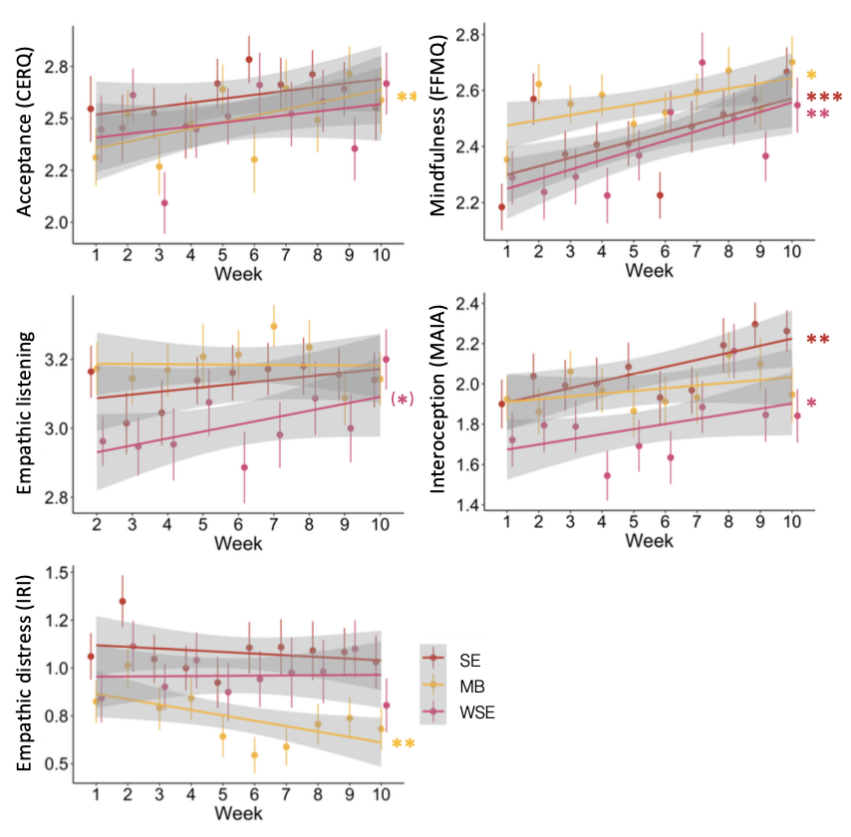
Trajectories of weekly assessed variables over the course of the intervention period in the socioemotional (SE), waitlist socioemotional (WSE), and mindfulness-based (MB) intervention groups. Significance level of (*) .10>*P*>.05, *P=.05, **P=.05, and ***P=.001. CERQ: Cognitive Emotion Regulation Questionnaire; FFMQ: Five Facet Mindfulness Questionnaire; IRI: Interpersonal Reactivity Index; MAIA: Multidimensional Assessment of Interoceptive Awareness.

Longitudinal changes in acceptance, empathic listening, empathic distress, mindfulness, and interoceptive awareness were modeled separately for the first (socioemotional and mindfulness-based) and second (waitlist socioemotional) intervention phases (Figure 5). In the first intervention phase, acceptance (β*_mindfulness-based_*=.04; *P*=.002) and mindfulness (β*_mindfulness-based_*=.02; *P*=.02) increased significantly in the mindfulness-based group over the course of the 10 weeks of the intervention, whereas empathic distress decreased over time (β*_mindfulness-based_*=−.03; *P*=.009). Empathic listening (β*_mindfulness-based_*=.00; *P*=.48) and interoceptive awareness (β*_mindfulness-based_*=.01; *P*=.11) showed no change during the first intervention period in the mindfulness-based group. In the socioemotional group, there was a significant increase in interoceptive awareness (β*_socioemotional_*=.04; *P*=.003) and mindfulness (β*_socioemotional_*=.03; *P*<.001). No change was found for acceptance (β*_socioemotional_*=.02; *P*=.10), empathic listening (β*_socioemotional_*=.01; *P*=.26), and empathic distress (β*_socioemotional_*=−.01; *P*=.72). None of the interaction terms between intervention group and time reached statistical significance (*P*>.05). In the waitlist socioemotional group, there was a significant increase in mindfulness (β*_waitlist socioemotional_*=.03; *P*=.002) and interoceptive awareness (β*_waitlist socioemotional_*=.03; *P*=.02) and a trend toward an increase in empathic listening, which was not significant (β*_waitlist socioemotional_*=.02; *P*=.05). No change over time was found in acceptance (β*_waitlist socioemotional_*=.02; *P*=.29) and empathic distress (β*_waitlist socioemotional_*=.01; *P*=.63).

Mediation models showed no (moderated) mediation effects of the slopes of acceptance, empathic listening, empathic distress, mindfulness, or interoceptive awareness on changes from preintervention assessment to postintervention assessment 1 in other-related compassion, behavioral empathy and compassion (EmpaToM), or empathic concern (IRI; Multimedia Appendix 8). In addition, no association between those outcome measures at postintervention assessment 1 and slopes (ie, b-paths) or moderated b-paths by intervention group was found (Multimedia Appendix 9). In the mindfulness-based group, the increase in self-compassion from preintervention assessment to postintervention assessment 1 was found to be mediated by a decrease in empathic distress, with an indirect effect of 0.07 (0.02, 0.14). A decrease in empathic distress could indeed predict levels of self-compassion at postintervention assessment 1 in both the socioemotional and mindfulness-based intervention groups (β=−9.17, 95% CI −15.59 to −3.11). In contrast, an increase in acceptance could predict self-compassion at postintervention assessment 1 only in the socioemotional group (β=6.63, 95% CI 0.52-12.38). In the waitlist socioemotional intervention group, no mediation or regression effects were found for the slopes of acceptance, empathic listening, empathic distress, mindfulness, or interoceptive awareness on changes from postintervention assessment 1 to postintervention assessment 2 (Multimedia Appendices 10 and 11).

## Discussion

### Principal Findings

The main aim of this RCT was to compare 2 daily app-delivered practices—a solitary mindfulness-based practice and a partner-based socioemotional practice, the Affect Dyad [19]—in their efficacy in boosting empathy and compassion for self and others using a multimethod approach (see Multimedia Appendix 12 for details on standardized reporting of this RCT). The study was embedded in a longitudinal mental health project during the COVID-19 pandemic in a large community sample [20], which was initially randomly recruited through the Berlin registration office.

Indeed, we found evidence that daily 12-minute app-delivered practice over 10 weeks could increase empathy on the EmpaToM task and self- and other-related compassion in a variety of questionnaires. Meanwhile, cognitive perspective-taking abilities (eg, ToM) did not change from before to after the intervention, indicating divergent validity of socioaffective and sociocognitive measures and their differential trainability through contemplative practices. Furthermore, the findings speak to differential intensities in practice effects on self-report and more objective computer task measures, with the latter producing weaker effects. Finally, we found differential psychological processes underlying the 2 interventions, specifically with regard to the development of self-compassion—training-related changes were mediated by a decrease in empathic distress in the mindfulness-based group and were associated with an increase in acceptance in the socioemotional dyad group.

### Distinction and Assessment of Compassion Toward the Self and Others

On the basis of a conceptual distinction between compassion toward oneself [39] and compassion as a motivation directed toward the welfare of others [9,26], we used a data-driven strategy to identify whether such a division is empirically supported in the CovSocial community sample of Berliners. Using a factor analytical approach with items from common compassion scales such as the SSCS-L [73], Sussex-Oxford Compassion Scales [43], and FoC [42], indeed 2 factors emerged that can be interpreted as reflective of self- and other-related compassion. The approach of aggregating several compassion scales into 2 distinct composites for self- and other-related compassion led to more reliable outcome measures and further allowed for the testing of potential differential effects of both trainings on different aspects of compassion.

Interestingly, only the subscales of self-kindness and self-judgment of the SSCS-L, a widely used measure of self-compassion, showed relevant loadings on the self-compassion factor. Neff [80] conceptualized self-compassion in 3 dimensions, namely, showing kindness toward oneself, understanding that suffering is part of a shared human experience, and being mindful of one’s own experiences. The 2 subscales integrated into the composite of self-compassion in this study refer to the dimension of self-kindness in a bipolar manner. Although mindfulness is an integral part of the definition of self-compassion by Neff [80], it was not found to be substantially related to the other measures of self-compassion included in this study. Similarly, items that measured the experience of common humanity, which is a part of both the SSCS-L and SOCS-S, did not show relevant loadings on the self-compassion factor, indicating a more distinct aspect of the concept.

### Practice Effects on Empathy and Compassion

Indeed, we observed significant training-related improvements on both aspects of compassion in all 3 intervention groups (socioemotional, mindfulness-based, and waitlist socioemotional), with effects being slightly higher for the self-compassion composite (Cohen *d*=0.30-0.44) than for the other-related compassion composite (Cohen *d*=0.24-0.35). Similarly, previous literature reviews and meta-analyses have highlighted that many mindfulness and compassion trainings specifically foster self-compassion [44,50], whereas results on other-related compassion have been less consistent [50]. However, previous studies have disproportionally focused on measuring self-compassion as a training target and less on compassion toward others [44,45]. Thus, based on the inclusion of self- and other-related aspects of compassion in this study, our results more systematically support that, although both aspects can be trained with mindfulness-based as well as dyadic socioemotional practice, there seems to be a slight advantage of such trainings in boosting self-compassion.

On the ecologically valid EmpaToM measures [49], we observed a slightly different pattern of mental training effects. First, and in line with our hypotheses, we found considerable effects only on socioaffective measures (empathy and compassion) but no change in the ToM measure. This is in line with previous findings on practice-specific effects on social skills [4]. Accordingly, perspective-taking can improve through mental practice that is specifically designed to target sociocognitive skills, such as the Perspective Dyad [19]. Furthermore, and again in accordance with our hypotheses, we observed the effects of both interventions on empathy. More specifically, both interventions were found to buffer a general decrease observed in the waitlist control group. The effects on compassion remained below the threshold of statistical significance, but the observed increase was of considerable size for both dyad intervention groups. This replicates previous findings of an increase in compassion on the EmpaToM after 3 months of intense in-person socioemotional but not other types of practice [4]. The small effect sizes of change in task-based compassion (Cohen *d*=0.08-0.11) highlight the difference in the dose-response relationship on self-report and behavioral measures [81]. Future research including larger sample sizes and longer trainings will have to explore the necessary statistical power and right dose of app-delivered training to bring about robust behavior changes.

When testing the validity of our findings using a frequently used empathy trait measure, the IRI [51], our findings also revealed no significant change in empathic concern, which is in contrast to the increase in the other-related compassion composite. However, similar to the results on the EmpaToM compassion ratings, there was a slight increase in empathic concern after 10 weeks of Affect Dyad training in the waitlist socioemotional group. The lack of change sensitivity of the IRI might be due to its rather trait-like nature (eg, “I would describe myself as a pretty soft-hearted person.”). Furthermore, the empathic concern scale is not per se a direct compassion scale, although it is related in scope. With regard to sociocognitive skills, similar to the results on ToM (EmpaToM) and in line with our hypotheses, the perspective-taking subscale of the IRI showed no training-related change.

Interestingly, both self-report and behavioral measures revealed a general decline in empathy and other-related compassion in the waitlist control group. With preintervention assessments in August 2021 and postintervention assessments in November 2021, this might be reflective of seasonal rhythms in affect that relate to changes in day length [82]. However, self-compassion remained stable in the control group. Alternatively, decreasing levels of empathy and compassion might be explained by an increase in compassion fatigue during the ongoing COVID-19 pandemic [29]. Compassion fatigue is characterized by a reduced capacity for compassion because of extended exposure to others’ negative feelings. However, evidence of affected empathy and compassion during the pandemic is somewhat inconsistent [83].

### Psychological Processes Underlying Practice Effects

With regard to the potential underlying psychological processes of the observed practice effects, we indeed found changes in various weekly measured variables during the 10-week intervention periods. Specifically, taking each intervention group alone, mindfulness-based practice significantly decreased empathic distress and increased acceptance and mindfulness over the 10 weeks of training, whereas the partner-based Affect Dyad significantly increased interoceptive awareness and mindfulness in both the socioemotional and waitlist socioemotional groups and increased empathic listening on trend in the waitlist socioemotional group, yet not statistically significant.

Furthermore, mediation analyses revealed that a decrease in empathic distress mediated the observed increase in self-compassion after mindfulness-based training. It has long been argued that mindfulness training can enable individuals to better regulate their emotions and become more equanimous and less reactive to external emotional triggers [60]. Thus, the effects of mindfulness practice on various outcomes might indeed rely on improvements in emotion regulation and other self-related processes [54,58]. In particular, equanimity has been proposed as a key factor in mediating mindfulness effects on compassion by developing a nonjudgmental attitude toward emotions [60]. However, interestingly, we found that decreases in empathic distress specifically mediated changes in self-compassion but not in compassion toward others. Although this result supports a distinction between developing self- and other-related compassion, the generally stronger effects on self-compassion and fatigue effects on other-related compassion in the waitlist control group might also account for it.

In contrast to the effects of mindfulness-based training and our expectations, empathic distress did not decrease during socioemotional dyadic training. A reduction in empathic distress is certainly a desirable training target, particularly in the context of the COVID-19 pandemic [29,30]. As Affect Dyad cultivates empathic listening, sharing difficult emotions may alleviate distress in the speaker but may elicit some empathic distress in the listener, especially in the case of intense emotions [84]. However, note that weekly coaching sessions provided instructions for the listener on how to engage in empathic listening and prevent distress. In line with this, we observed a nonsignificant trend of increased empathic listening in the waitlist socioemotional group and no increase in empathic distress.

However, more importantly, and in line with our hypotheses, we observed that an increase in acceptance could differentially predict levels of self-compassion at postintervention assessment in the socioemotional group compared with the mindfulness-based intervention group. Thus, cultivating acceptance of difficult emotions and gratitude in daily life through the Affect Dyad can foster a care system that relates not only to feelings of nurturing and love for others [65,85] but also to self-love and self-compassion.

### Limitations

One of the limitations of the reported study relates to technical issues that were encountered with the smartphone app that was used for daily practice. During the first few weeks of the first intervention phase, in 25.42% (412/1621) of cases, a stable internet connection could not be established at the first attempt between dyadic partners in the socioemotional training. This might have negatively affected both motivation for and attitude toward daily practice of the Affect Dyad and, thus, caused the higher dropout rate in the socioemotional group (24/95, 25%) than in the mindfulness-based group (16/97, 16%). However, on average, compliance in form of performed daily practice was higher in both socioemotional intervention groups (socioemotional: 88.79%, SD 8.15%; waitlist socioemotional: 90.19%, SD 7.24%) than in the mindfulness-based training (86.4%, SD 14.53%; *P*<.001). Future studies could help understand whether higher compliance for partner-based over solitary practice is related to social norms given its interpersonal modality.

Despite sample recruitment in accordance with a priori power and sample size calculations, there was a high rate of participant dropout as early as between study onboarding and preintervention assessment (a total of 32/285, 11.2%). As the recruited sample was naive to contemplative practice (note that previous experience was an exclusion criterion), they might have reconsidered participation once introduced to the practice content and requirements of daily training. However, this rather heterogeneous community sample of a COVID-19 mental health study is also an asset with regard to the representativeness of the population. The findings are promising that the 2 app-delivered trainings can indeed be used to boost empathy and compassion in the general population.

### Comparison With Prior Work

Previous research has suggested an advantage of socioemotional contemplative training, including loving-kindness meditation, on compassion [4,48]. However, these findings were based on an extensive training study that combined several training approaches [41]. For the first time, this RCT selectively probed the efficacy of the app-delivered partner-based Affect Dyad in comparison with mindfulness-based practice. The novelty of this study also relates to the modality of the Affect Dyad. Thus, findings are still scarce on positive effects of partner-based contemplative practice on socioaffective capacities [53]. Indeed, the 10-week low-dose web-based practice with the Affect Dyad in this study could replicate the effects of the 3-month Affect module in the ReSource project in increasing empathy and compassion [4,48].

### Conclusions

In this RCT, we showed that app-delivered, mindfulness-based, and partner-based socioemotional practices can promote empathy and compassion for self and others. Thereby, the effects were stronger for state self-report measures than for task-based or dispositional measures, pointing to a lower dose-response relationship in those markers. In addition, we found differential mechanisms for practice effects on self-compassion. Although mindfulness practice takes effect via a decrease in empathic distress, changes in self-compassion after Affect Dyad training were associated with an increase in acceptance, which might be related to a more general activation of a motivational human care system. In sum, the findings support that app-delivered trainings such as these could be a promising scalable solution to boost social skills and, thus, mitigate the threats and challenges to social cohesion, mental health, and psychological well-being faced by societies worldwide.

## Appendix

Multimedia Appendix 1 Mental training intervention protocol for CovSocial phase 2: summary.

Multimedia Appendix 2 A priori sample size calculation.

Multimedia Appendix 3 CovSocial phase 2 sample demographics compared with phase 1 sample and the Berlin general population.

Multimedia Appendix 4 CovSocial smartphone app.

Multimedia Appendix 5 Descriptive statistics for theory of mind and factual reasoning split by measurement occasion and group.

Multimedia Appendix 6 Correlations between behavioral and self-report measures.

Multimedia Appendix 7 Mean plots of the empathic concern and perspective taking subscales of the Interpersonal Reactivity Index in the socioemotional, waitlist socioemotional, and mindfulness-based intervention groups and the waitlist control group and group differences in intervention-related change. Means and SEs; significance level of (*) .10>α>.05.

Multimedia Appendix 8 Indirect moderated mediation effects of slopes on change in outcome variables in the socioemotional and mindfulness-based mental trainings.

Multimedia Appendix 9 Regression estimates of mediator slopes on outcome measures and group-moderated regressions.

Multimedia Appendix 10 Indirect mediation effects of slopes on changes in outcome variables in the waitlist socioemotional training.

Multimedia Appendix 11 Regression estimates of mediator slopes on outcome measures in the waitlist socioemotional mental training.

Multimedia Appendix 12 Consolidated Standards of Reporting Trials (CONSORT) checklist.

## Abbreviations

FoC: Fears of Compassion Scales
IRI: Interpersonal Reactivity Index
LMM: linear mixed-effects model
RCT: randomized controlled trial
RMSEA: root mean square error of approximation
SOCS-S: Sussex-Oxford Compassion for the Self Scale
SSCS-L: long form of the State Self-Compassion Scale
TLI: Tucker-Lewis index
ToM: theory of mind

## Data Availability

The raw data sets generated and analyzed during this study are not publicly available because of proprietary rights and data protection policies but are available from the corresponding author upon reasonable request.
