# Supplementary material for: Boosting Empathy and Compassion Through Mindfulness-Based and Socioemotional Dyadic Practice: Randomized Controlled Trial With App-Delivered Trainings
Source: J Med Internet Res. 2023 Jul 26;25:e45027. doi: 10.2196/45027 (PMC10413229; doi:10.2196/45027)
Supplement: Multimedia Appendix 2 [file jmir_v25i1e45027_app2.docx]

**A priori sample size calculation**

A priori effect size and power calculations were conducted for repeated measures analyses of variance including interaction terms of between- and within-group contrasts using G*Power [71]. A significance level *α* = .05 and expected power of .80 were applied in a 3 (groups) x 2 (timepoints) design. The estimate for repeated measures correlation was constrained to the lowest retest reliability of behavioral or biomarkers assessed in the project (i.e., Cortisol Awakening Response; *r* = .39). Besides, small effect sizes (*f* = .10) were assumed due to previous findings from the ReSource project [41], a large-scale intervention study that entailed core exercises of mental training programs included in the current study. This resulted in a calculated sample size of *n* = 297.
