# Supplementary material for: Boosting Empathy and Compassion Through Mindfulness-Based and Socioemotional Dyadic Practice: Randomized Controlled Trial With App-Delivered Trainings
Source: J Med Internet Res. 2023 Jul 26;25:e45027. doi: 10.2196/45027 (PMC10413229; doi:10.2196/45027)
Supplement: Multimedia Appendix 3 [file jmir_v25i1e45027_app3.docx]

CovSocial phase 2 sample demographics compared to phase 1 sample and Berlin general population

|  |  | Phase 2 sample | Rest of  Phase 1 sample | Berlin population |
| --- | --- | --- | --- | --- |
|  |  | n = 285 | n = 3,238 | n = 3,669,491 |
|  |  | M ± SD  n (%) | M ± SD  n (%) | M  n (%) |
|  |  |  |  |  |
| Age |  |  |  |  |
|  |  | 44.12 ± 11.39 | 43.93 ± 12.79 | 43 |
| Sex |  |  |  |  |
|  | male | 67 (23.5%) | 1,162 (35.9%) | 49.5% |
|  | female | 218 (76.5%) | 2,076 (64.1%) | 50.5% |
| Marital status |  |  |  |  |
|  | single | 182 (63.9%) | 1,957 (60.4%) | 65.8% |
|  | married / cohabiting | 103 (36.1%) | 1,281 (39.6%) | 34.2% |
| Years of education |  |  |  |  |
|  |  | 17.85 ± 3.59 | 16.97 ± 3.88 |  |
| Employment status |  |  |  |  |
|  | full time / part-time | 245 (86.0%) | 2662 (82.2%) | 89.5% |
|  | none | 40 (14.0%) | 576 (17.8%) | 10.5% |
| Household income |  |  |  |  |
|  | > average^1^ | 179 (62.8%) | 1,896 (58.6%) | 50% |
|  | < average^1^ | 105 (36.8%) | 1,290 (39.8%) | 50% |
|  | NA | 1 (0.4%) | 52 (1.6%) |  |
| Lifetime prevalence of mental disorder |  |  |  |  |
|  | yes | 57 (20.0%) | 819 (25.0%) |  |
|  | no | 226 (79.3%) | 2,326 (72.0%) |  |
|  | NA | 2 (0.7%) | 93 (3.0%) |  |

*Note.* ^1^ The average monthly net income in Berlin is approximately 2,175€ [72]
