# Supplementary material for: Boosting Empathy and Compassion Through Mindfulness-Based and Socioemotional Dyadic Practice: Randomized Controlled Trial With App-Delivered Trainings
Source: J Med Internet Res. 2023 Jul 26;25:e45027. doi: 10.2196/45027 (PMC10413229; doi:10.2196/45027)
Supplement: Multimedia Appendix 4 [file jmir_v25i1e45027_app4.docx]

**CovSocial smartphone app**

Both interventions were delivered through the CovSocial mobile- and web-app. The CovSocial app is a research tool which was developed by CosmoCode GmbH for the Max Planck Society, and was designed as extension of an app that has already been used in the ReSource project. At the end of the pretest, participants in SE and MB groups received an introduction to the installation, default settings and functions of the CovSocial app. Similar introduction was given to participants in the WSE group at the end of posttest 1.
