# Supplementary material for: Boosting Empathy and Compassion Through Mindfulness-Based and Socioemotional Dyadic Practice: Randomized Controlled Trial With App-Delivered Trainings
Source: J Med Internet Res. 2023 Jul 26;25:e45027. doi: 10.2196/45027 (PMC10413229; doi:10.2196/45027)
Supplement: Multimedia Appendix 5 [file jmir_v25i1e45027_app5.docx]

Descriptive statistics for theory of mind (ToM) and factual reasoning split by measurement occasion and group.

|  |  | Pretest | | Posttest 1 | | Posttest 2 | |
| --- | --- | --- | --- | --- | --- | --- | --- |
|  | Group | M | SD | M | SD | M | SD |
| ToM  Composite |  |  |  |  |  |  |  |
|  | SE | 0.11 | 0.75 | 0.09 | 0.74 |  |  |
|  | MB | 0.05 | 0.65 | –0.05 | 0.60 |  |  |
|  | WC | 0.05 | 0.74 | –0.02 | 0.70 |  |  |
|  | WSE |  |  |  |  | –0.09 | 0.82 |
| ToM  Accuracy |  |  |  |  |  |  |  |
|  | SE | 0.52 | 0.18 | 0.56 | 0.17 |  |  |
|  | MB | 0.55 | 0.16 | 0.58 | 0.15 |  |  |
|  | WC | 0.53 | 0.16 | 0.56 | 0.15 |  |  |
|  | WSE |  |  |  |  | 0.55 | 0.18 |
| ToM  Response time |  |  |  |  |  |  |  |
|  | SE | 8.16 | 1.37 | 8.39 | 1.33 |  |  |
|  | MB | 8.18 | 1.38 | 8.10 | 1.25 |  |  |
|  | WC | 8.04 | 1.39 | 8.07 | 1.24 |  |  |
|  | WSE |  |  |  |  | 7.86 | 1.43 |
| Factual reasoning Composite |  |  |  |  |  |  |  |
|  | SE | 0.06 | 0.76 | 0.05 | 0.81 |  |  |
|  | MB | 0.05 | 0.75 | –0.09 | 0.74 |  |  |
|  | WC | 0.02 | 0.72 | –0.04 | 0.78 |  |  |
|  | WSE |  |  |  |  | –0.23 | 0.78 |
| Factual reasoning Accuracy |  |  |  |  |  |  |  |
|  | SE | 0.59 | 0.18 | 0.62 | 0.19 |  |  |
|  | MB | 0.60 | 0.18 | 0.63 | 0.19 |  |  |
|  | WC | 0.58 | 0.20 | 0.62 | 0.19 |  |  |
|  | WSE |  |  |  |  | 0.64 | 0.18 |
| Factual reasoning Response time |  |  |  |  |  |  |  |
|  | SE | 8.52 | 1.27 | 8.72 | 1.28 |  |  |
|  | MB | 8.55 | 1.35 | 8.39 | 1.16 |  |  |
|  | WC | 8.36 | 1.27 | 8.43 | 1.22 |  |  |
|  | WSE |  |  |  |  | 8.08 | 1.30 |

*Note.* SE/WSE = (waitlist) socio-emotional intervention, MB = mindfulness-based intervention, WC = waitlist control
