# Supplementary material for: Boosting Empathy and Compassion Through Mindfulness-Based and Socioemotional Dyadic Practice: Randomized Controlled Trial With App-Delivered Trainings
Source: J Med Internet Res. 2023 Jul 26;25:e45027. doi: 10.2196/45027 (PMC10413229; doi:10.2196/45027)
Supplement: Multimedia Appendix 6 [file jmir_v25i1e45027_app6.docx]

#### **Correlations between behavioral and self-report measures**

Correlation analyses were conducted to compare computer task measures of compassion and ToM (EmpaToM; [49]), composites for self-compassion and compassion towards others, and self-report measures of empathic concern and perspective taking (IRI; [51]).

Despite their differences in change sensitivity in our study, all measures of compassion towards others, that is, the composite of self-report scales, the EmpaToM task measure, and the IRI subscale for empathic concern, were moderately correlated with each other at study pretest (*r* = .35 to .53). Socio-cognitive measures of ToM (EmpaToM) and perspective taking (IRI) did not show any correlation at study pretest. This corroborates prevailing doubts about the conceptual overlap of self-report measures of socio-cognitive skills and ToM measured with a behavioral task paradigm.


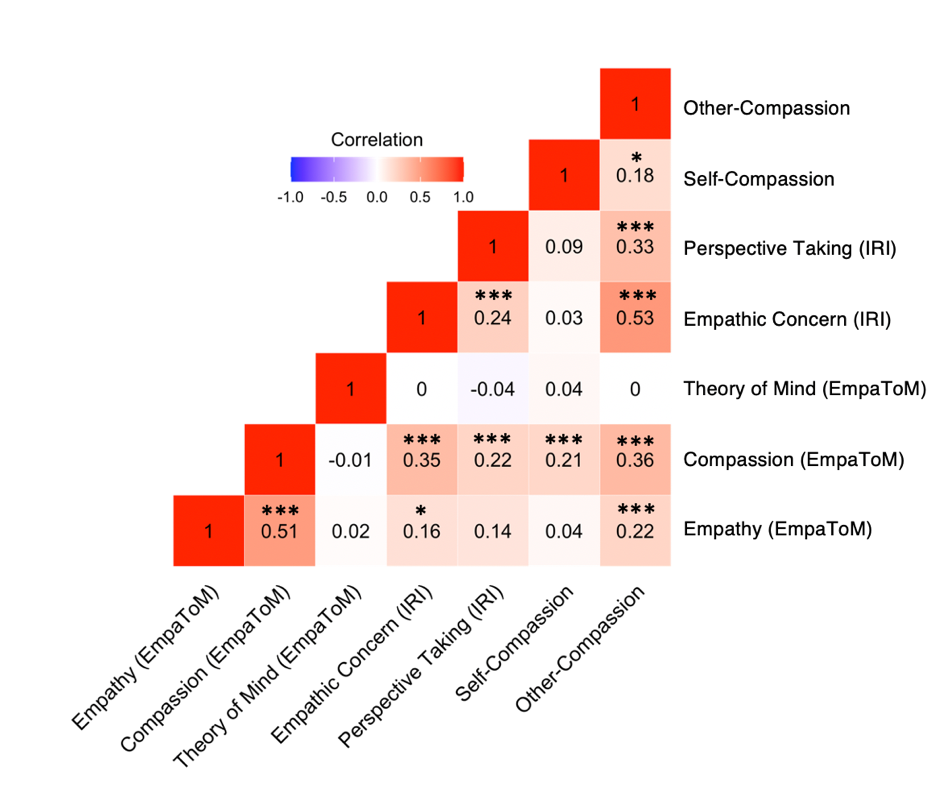


**Figure.** Spearman correlations of pretest scores on empathy, compassion and theory of mind in a computer-based task (EmpaToM), empathic concern and perspective taking subscales of the Interpersonal Reactivity Index (IRI), and questionnaire composites of self-compassion and other-compassion. Significance level of **𝛼* = .05 and ****𝛼* = .001.
