# Supplementary material for: Boosting Empathy and Compassion Through Mindfulness-Based and Socioemotional Dyadic Practice: Randomized Controlled Trial With App-Delivered Trainings
Source: J Med Internet Res. 2023 Jul 26;25:e45027. doi: 10.2196/45027 (PMC10413229; doi:10.2196/45027)
Supplement: Multimedia Appendix 7 [file jmir_v25i1e45027_app7.docx]

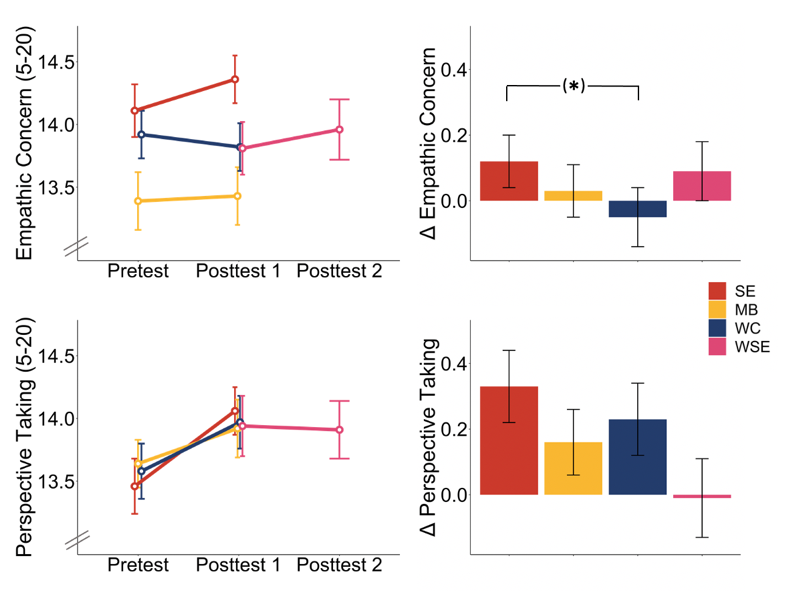


Mean plots of subscales Empathic Concern and Perspective Taking of the Interpersonal Reactivity Index in the socio-emotional (SE, WSE) and mindfulness-based (MB) intervention groups and the waitlist control (WC) group, and group differences in intervention-related change (B). Means and standard errors; significance level of ^(^*^)^ .10 > *𝛼* > .05.
