# Supplementary material for: Boosting Empathy and Compassion Through Mindfulness-Based and Socioemotional Dyadic Practice: Randomized Controlled Trial With App-Delivered Trainings
Source: J Med Internet Res. 2023 Jul 26;25:e45027. doi: 10.2196/45027 (PMC10413229; doi:10.2196/45027)
Supplement: Multimedia Appendix 8 [file jmir_v25i1e45027_app8.docx]

Indirect moderated mediation effects of slopes on change in outcome variables in the socio-emotional (SE) and mindfulness-based (MB) mental training.

|  |  | Group | Effect | se | CI_LL_ | CI_UL_ |
| --- | --- | --- | --- | --- | --- | --- |
|  |  |  |  |  |  |  |
| **Self-Compassion** |  |  |  |  |  |  |
|  | Acceptance | MB | 0.00 | 0.01 | –0.01 | 0.01 |
|  |  | SE | 0.01 | 0.02 | –0.03 | 0.06 |
|  | Empathic distress | MB | 0.07 | 0.03 | 0.02 | 0.14 |
|  |  | SE | 0.04 | 0.03 | –0.00 | 0.10 |
|  | Interoception | MB | –0.00 | 0.01 | –0.02 | 0.01 |
|  |  | SE | –0.00 | 0.01 | –0.02 | 0.01 |
|  | Mindfulness | MB | –0.01 | 0.01 | –0.04 | 0.01 |
|  |  | SE | –0.02 | 0.02 | –0.06 | 0.01 |
|  | Empathic listening | MB | 0.00 | 0.01 | –0.01 | 0.02 |
|  |  | SE | 0.00 | 0.01 | –0.01 | 0.02 |
| **Other-Compassion** |  |  |  |  |  |  |
|  | Acceptance | MB | 0.01 | 0.01 | –0.01 | 0.03 |
|  |  | SE | 0.01 | 0.01 | –0.01 | 0.03 |
|  | Empathic distress | MB | 0.01 | 0.02 | –0.02 | 0.05 |
|  |  | SE | –0.01 | 0.02 | –0.05 | 0.02 |
|  | Interoception | MB | –0.00 | 0.01 | –0.04 | 0.03 |
|  |  | SE | –0.01 | 0.02 | –0.06 | 0.03 |
|  | Mindfulness | MB | 0.00 | 0.01 | –0.01 | 0.02 |
|  |  | SE | 0.01 | 0.02 | –0.02 | 0.05 |
|  | Empathic listening | MB | 0.00 | 0.01 | –0.01 | 0.01 |
|  |  | SE | –0.00 | 0.01 | –0.03 | 0.02 |
| **Compassion (EmpaToM)** |  |  |  |  |  |  |
|  | Acceptance | MB | –0.00 | 0.00 | –0.01 | 0.01 |
|  |  | SE | –0.00 | 0.01 | –0.03 | 0.02 |
|  | Empathic distress | MB | 0.00 | 0.01 | –0.01 | 0.04 |
|  |  | SE | –0.01 | 0.01 | –0.03 | 0.01 |
|  | Interoception | MB | –0.02 | 0.01 | –0.05 | 0.01 |
|  |  | SE | –0.00 | 0.03 | –0.05 | 0.06 |
|  | Mindfulness | MB | –0.00 | 0.01 | –0.02 | 0.01 |
|  |  | SE | –0.00 | 0.01 | –0.03 | 0.02 |
|  | Empathic listening | MB | –0.01 | 0.01 | –0.02 | 0.01 |
|  |  | SE | 0.01 | 0.02 | –0.01 | 0.06 |
| **Empathy (EmpaToM)** |  |  |  |  |  |  |
|  | Acceptance | MB | –0.00 | 0.00 | –0.01 | 0.01 |
|  |  | SE | –0.00 | 0.01 | –0.03 | 0.01 |
|  | Empathic distress | MB | 0.01 | 0.01 | –0.01 | 0.04 |
|  |  | SE | 0.00 | 0.01 | –0.02 | 0.02 |
|  | Interoception | MB | –0.00 | 0.00 | –0.01 | 0.01 |
|  |  | SE | 0.01 | 0.01 | –0.02 | 0.04 |
|  | Mindfulness | MB | 0.00 | 0.01 | –0.01 | 0.01 |
|  |  | SE | –0.00 | 0.01 | –0.03 | 0.02 |
|  | Empathic listening | MB | 0.00 | 0.01 | –0.01 | 0.01 |
|  |  | SE | 0.00 | 0.01 | –0.01 | 0.02 |
| **Empathic concern (IRI)** |  |  |  |  |  |  |
|  | Acceptance | MB | 0.00 | 0.01 | –0.01 | 0.02 |
|  |  | SE | –0.00 | 0.01 | –0.03 | 0.02 |
|  | Empathic distress | MB | 0.00 | 0.00 | –0.01 | 0.01 |
|  |  | SE | 0.00 | 0.01 | –0.01 | 0.01 |
|  | Interoception | MB | –0.00 | 0.01 | –0.02 | 0.02 |
|  |  | SE | 0.01 | 0.02 | –0.02 | 0.05 |
|  | Mindfulness | MB | 0.00 | 0.01 | –0.01 | 0.02 |
|  |  | SE | –0.01 | 0.01 | –0.04 | 0.01 |
|  | Empathic listening | MB | –0.01 | 0.01 | –0.03 | 0.01 |
|  |  | SE | –0.00 | 0.01 | –0.02 | 0.01 |
