# Supplementary material for: Boosting Empathy and Compassion Through Mindfulness-Based and Socioemotional Dyadic Practice: Randomized Controlled Trial With App-Delivered Trainings
Source: J Med Internet Res. 2023 Jul 26;25:e45027. doi: 10.2196/45027 (PMC10413229; doi:10.2196/45027)
Supplement: Multimedia Appendix 9 [file jmir_v25i1e45027_app9.docx]

Regression estimates of mediator slopes on outcome measures, and group-moderated regressions.

| Outcome | Predictor | Coefficient | se | CI_LL_ | CI_UL_ |
| --- | --- | --- | --- | --- | --- |
|  |  |  |  |  |  |
| **Self-Compassion** |  |  |  |  |  |
|  | Acceptance | 0.07 | 1.54 | –2.94 | 3.16 |
|  | Empathic distress | –9.17 | 3.17 | –15.59 | –3.11 |
|  | Interoception | 1.35 | 1.17 | –.92 | 3.71 |
|  | Mindfulness | 1.41 | 1.61 | –1.64 | 4.73 |
|  | Empathic listening | –2.20 | 1.56 | –5.17 | 1.01 |
|  | Acceptance x SE | 6.63 | 3.03 | 0.52 | 12.38 |
|  | Empathic distress x SE | 3.98 | 4.24 | –4.58 | 12.14 |
|  | Interoception x SE | –0.21 | 2.28 | –4.91 | 4.12 |
|  | Mindfulness x SE | 1.90 | 2.59 | –3.23 | 7.04 |
|  | Empathic listening x SE | 0.45 | 2.64 | –4.89 | 5.66 |
| **Other-Compassion** |  |  |  |  |  |
|  | Acceptance | 2.30 | 1.76 | –0.58 | 6.44 |
|  | Empathic distress | –2.76 | 3.34 | –9.55 | 3.64 |
|  | Interoception | –0.40 | 1.53 | –3.60 | 2.46 |
|  | Mindfulness | 0.75 | 1.95 | –2.91 | 4.76 |
|  | Empathic listening | 0.35 | 1.37 | –2.15 | 3.20 |
|  | Acceptance x SE | 0.36 | 2.97 | –5.42 | 6.17 |
|  | Empathic distress x SE | 5.07 | 4.52 | –3.48 | 14.16 |
|  | Interoception x SE | –0.62 | 2.61 | –5.91 | 4.34 |
|  | Mindfulness x SE | 3.91 | 3.47 | –3.01 | 10.56 |
|  | Empathic listening x SE | –1.25 | 2.86 | –7.13 | 4.27 |
| **Compassion**  **(EmpaToM)** |  |  |  |  |  |
|  | Acceptance | –0.07 | 0.94 | –1.91 | 1.78 |
|  | Empathic distress | –2.96 | 1.82 | –6.58 | 0.56 |
|  | Interoception | –1.00 | 0.80 | –2.64 | 0.52 |
|  | Mindfulness | –0.40 | 1.13 | –2.63 | 1.78 |
|  | Empathic listening | 0.65 | 0.88 | –1.13 | 2.31 |
|  | Acceptance x SE | –1.79 | 2.11 | –5.83 | 2.43 |
|  | Empathic distress x SE | 4.90 | 2.74 | –0.57 | 10.17 |
|  | Interoception x SE | 0.98 | 1.73 | –2.21 | 4.60 |
|  | Mindfulness x SE | –0.47 | 2.08 | –4.68 | 3.63 |
|  | Empathic listening x SE | –2.40 | 1.87 | –6.15 | 1.20 |
| **Empathy**  **(EmpaToM)** |  |  |  |  |  |
|  | Acceptance | 0.12 | 1.38 | –2.73 | 2.71 |
|  | Empathic distress | 4.95 | 2.79 | –0.65 | 10.28 |
|  | Interoception | 0.06 | 1.04 | –1.98 | 2.09 |
|  | Mindfulness | –1.14 | 1.33 | –3.84 | 1.36 |
|  | Empathic listening | –0.28 | 1.53 | –3.24 | 2.75 |
|  | Acceptance x SE | 1.36 | 2.98 | –4.85 | 7.06 |
|  | Empathic distress x SE | –4.55 | 3.98 | –11.90 | 3.35 |
|  | Interoception x SE | –3.32 | 2.15 | –7.55 | 1.01 |
|  | Mindfulness x SE | 4.20 | 2.89 | –1.16 | 10.21 |
|  | Empathic listening x SE | –0.09 | 2.65 | –5.46 | 4.90 |
| **Empathic concern**  **(IRI)** |  |  |  |  |  |
|  | Acceptance | 2.15 | 2.20 | –2.17 | 6.62 |
|  | Empathic distress | 0.05 | 3.63 | –6.55 | 7.54 |
|  | Interoception | –0.36 | 1.65 | –3.43 | 2.92 |
|  | Mindfulness | 1.51 | 1.91 | –2.18 | 5.37 |
|  | Empathic listening | 3.06 | 1.91 | –0.62 | 6.76 |
|  | Acceptance x SE | –2.45 | 4.36 | –11.03 | 6.03 |
|  | Empathic distress x SE | 2.04 | 5.35 | –8.87 | 12.30 |
|  | Interoception x SE | 2.35 | 3.41 | –4.39 | 9.09 |
|  | Mindfulness x SE | –4.28 | 3.38 | –11.29 | 2.18 |
|  | Empathic listening x SE | –0.39 | 3.22 | –6.51 | 6.17 |

*Note.* SE = dummy coded contrast between socio-emotional mental training and mindfulness-based mental training.
