# Supplementary material for: Boosting Empathy and Compassion Through Mindfulness-Based and Socioemotional Dyadic Practice: Randomized Controlled Trial With App-Delivered Trainings
Source: J Med Internet Res. 2023 Jul 26;25:e45027. doi: 10.2196/45027 (PMC10413229; doi:10.2196/45027)
Supplement: Multimedia Appendix 10 [file jmir_v25i1e45027_app10.docx]

Indirect mediation effects of slopes on change in outcome variables in the waitlist socio-emotional training.

|  |  | Effect | se | CI_LL_ | CI_UL_ |
| --- | --- | --- | --- | --- | --- |
|  |  |  |  |  |  |
| **Self-Compassion** |  |  |  |  |  |
|  | Acceptance | 0.01 | 0.03 | –0.03 | 0.08 |
|  | Empathic distress | 0.03 | 0.04 | –0.04 | 0.12 |
|  | Interoception | 0.02 | 0.04 | –0.06 | 0.10 |
|  | Mindfulness | 0.00 | 0.02 | –0.04 | 0.03 |
|  | Empathic listening | –0.01 | 0.02 | –0.06 | 0.03 |
| **Other-Compassion** |  |  |  |  |  |
|  | Acceptance | 0.00 | 0.02 | –0.05 | 0.04 |
|  | Empathic distress | 0.02 | 0.03 | –0.02 | 0.09 |
|  | Interoception | –0.01 | 0.02 | –0.07 | 0.03 |
|  | Mindfulness | 0.01 | 0.02 | –0.02 | 0.06 |
|  | Empathic listening | 0.00 | 0.03 | –0.05 | 0.06 |
| **Compassion (EmpaToM)** |  |  |  |  |  |
|  | Acceptance | –0.00 | 0.05 | –0.04 | 0.11 |
|  | Empathic distress | –0.03 | 0.03 | –0.11 | 0.02 |
|  | Interoception | 0.00 | 0.03 | –0.06 | 0.06 |
|  | Mindfulness | 0.00 | 0.02 | –0.04 | 0.05 |
|  | Empathic listening | 0.02 | 0.04 | –0.05 | 0.13 |
| **Empathy (EmpaToM)** |  |  |  |  |  |
|  | Acceptance | 0.00 | 0.05 | –0.11 | 0.04 |
|  | Empathic distress | –0.00 | 0.02 | –0.05 | 0.03 |
|  | Interoception | 0.00 | 0.03 | –0.07 | 0.07 |
|  | Mindfulness | –0.01 | 0.03 | –0.06 | 0.05 |
|  | Empathic listening | 0.00 | 0.02 | –0.02 | 0.05 |
| **Empathic concern (IRI)** |  |  |  |  |  |
|  | Acceptance | 0.00 | 0.02 | –0.04 | 0.05 |
|  | Empathic distress | –0.01 | 0.03 | –0.10 | 0.03 |
|  | Interoception | 0.01 | 0.02 | –0.02 | 0.06 |
|  | Mindfulness | 0.01 | 0.05 | –0.09 | 0.11 |
|  | Empathic listening | –0.00 | 0.02 | –0.05 | 0.03 |
