# Supplementary material for: Boosting Empathy and Compassion Through Mindfulness-Based and Socioemotional Dyadic Practice: Randomized Controlled Trial With App-Delivered Trainings
Source: J Med Internet Res. 2023 Jul 26;25:e45027. doi: 10.2196/45027 (PMC10413229; doi:10.2196/45027)
Supplement: Multimedia Appendix 11 [file jmir_v25i1e45027_app11.docx]

Regression estimates of mediator slopes on outcome measures in the waitlist socio-emotional mental training.

| Outcome | Predictor | Coefficient | se | CI_LL_ | CI_UL_ |
| --- | --- | --- | --- | --- | --- |
|  |  |  |  |  |  |
| **Self-Compassion** |  |  |  |  |  |
|  | Acceptance | 4.26 | 2.69 | –1.56 | 8.94 |
|  | Empathic distress | –4.86 | 4.86 | –14.08 | 5.29 |
|  | Interoception | 1.80 | 2.55 | –3.81 | 6.26 |
|  | Mindfulness | –0.94 | 2.13 | –4.94 | 3.55 |
|  | Empathic listening | 0.69 | 2.13 | –3.54 | 5.05 |
| **Other-Compassion** |  |  |  |  |  |
|  | Acceptance | –3.88 | 2.60 | –7.90 | 2.20 |
|  | Empathic distress | –8.36 | 4.20 | –16.32 | 0.37 |
|  | Interoception | –1.86 | 2.63 | –7.19 | 3.22 |
|  | Mindfulness | 1.78 | 2.19 | –2.78 | 5.84 |
|  | Empathic listening | 0.25 | 2.67 | –4.64 | 5.93 |
| **Compassion (EmpaToM)** |  |  |  |  |  |
|  | Acceptance | –0.73 | 1.87 | –4.29 | 3.11 |
|  | Empathic distress | 4.42 | 3.23 | –2.43 | 10.26 |
|  | Interoception | 3.20 | 1.93 | –0.79 | 6.79 |
|  | Mindfulness | 0.53 | 1.95 | –3.06 | 4.58 |
|  | Empathic listening | –5.06 | 2.19 | –9.22 | –0.50 |
| **Empathy (EmpaToM)** |  |  |  |  |  |
|  | Acceptance | -2.61 | 1.83 | –6.10 | 1.12 |
|  | Empathic distress | –0.54 | 3.49 | –6.97 | 6.72 |
|  | Interoception | –0.26 | 2.21 | –4.42 | 4.28 |
|  | Mindfulness | 0.72 | 1.74 | –2.78 | 4.12 |
|  | Empathic listening | 2.56 | 2.01 | –1.65 | 6.35 |
| **Empathic concern (IRI)** |  |  |  |  |  |
|  | Acceptance | –2.25 | 3.03 | –7.76 | 4.33 |
|  | Empathic distress | 4.79 | 6.15 | –8.12 | 16.30 |
|  | Interoception | 2.59 | 2.78 | –2.96 | 7.91 |
|  | Mindfulness | 8.88 | 2.95 | 3.57 | 15.15 |
|  | Empathic listening | –0.80 | 3.22 | –7.16 | 5.37 |
